# Amelioration of radiation-induced liver damage by *p*-coumaric acid in mice

**DOI:** 10.1007/s10068-022-01118-8

**Published:** 2022-06-15

**Authors:** Yun-Hong Li, Jiang-Xue Wu, Qian He, Jia Gu, Lin Zhang, Hao-Zhi Niu, Xin-Wen Zhang, Han-Ting Zhao, Jia-Ying Xu, Li-Qiang Qin

**Affiliations:** 1 Department of Nutrition and Food Hygiene, School of Public Health, Soochow University, 199 Renai Road, Suzhou, 215123 Jiangsu Province China; 2 State Key Laboratory of Radiation Medicine and Protection, School for Radiological and Interdisciplinary Sciences (RAD-X) Collaborative Innovation Center of Radiation Medicine of Jiangsu Higher Education Institutions, Soochow University, 199 Renai Road, Suzhou, 215123 Jiangsu Province China

**Keywords:** *p*-coumaric acid, Radiation, Liver damage, Protective effect

## Abstract

Radiation-induced liver damage (RILD) is a spiny problem in radiotherapy or other circumstances that exposure to radiation. The need for radioprotective agent is increasing to protect liver tissue. This study aimed to explore the hepatoprotective effect of *p*-coumaric acid (CA) against RILD. C57BL/6 male mice were exposed to 4 Gy irradiation and administrated with CA for 4 days starting on the same day of irradiation. Mice were sacrificed to obtain blood and liver tissues on day 3.5 or 14 post irradiation, respectively. The blood and liver tissues were collected. As compared with the only irradiated group, CA supplementation improved liver morphology, decreased serum alanine aminotransferase and aspartate aminotransferase, inhibited BCL2-associated X (BAX) protein expression, and improved the mice hematopoietic function. CA at the dose of 100 mg/kg body weight showed better effect compared to the other doses. Thus, CA might possess potential to protect against RILD.

## Introduction

Exposure to ionizing radiation (IR) occurs during radiotherapy, medical diagnosis, experimentation, work in nuclear environment, and accidental radiation releases (Radwan and Mohamed, 2018). Radiotherapy is commonly used in treatment for liver cancer, or other tumors in the upper abdomen or lower thorax, wherein liver is in the radiation area (Meydan et al., 2011). While, liver is a radio-sensitive organ that is susceptible to acute and chronic radiation injuries (Kjærgaard et al., 2020). After received a radiation dose exceeds 30 Gy, 5–10% patients may develop radiation-induced liver damage (RILD); with 43 Gy dose radiation, approximately 50% patients may develop RILD (Zhang et al., 2014). Synthetic compounds like lipoic acid, 2-mercaptopropionglycine and amifostine have been reported to be good radioprotectors, while high systemic toxicity limits their practical application (Das et al., 2014). Therefore, it is urgent to develop less or non-toxic compounds for radioprotection.

Bioactive compounds derived from plants have gained considerable attention for their anti-tumor and radioprotective properties (Bakshi et al., 2020). Polyphenols, widely spread throughout the plant kingdom, have the potential to be good radioprotectors that can alleviate IR-induced injury (Li et al., 2016). However, most phenolic acids showed low bioaccessibility in vivo, thus limited them to be used as anti-cancer agents and radio-protectors. The *p*-coumaric acid (CA), a polyphenol that possesses higher bioavailability, might be a good choice. In vitro digestion study showed that, as compared with other phenolic acids (including gallic acid, ferulic acid, chlorogenic acid and catechin), CA possessed the highest bioaccessibility among most tested food matrices (Sęczyk et al., 2020). In vivo study also showed that CA possessed a high bioaccessibility as compared with other phenolic acids in table olives (Kundisová et al., 2020).

CA is commonly found in fruits, vegetables and cereals. It exerts many beneficial effects including anti-oxidant, anti-inflammatory, anti-cancer, anti-diabetes, improve glucose homeostasis, neuroprotective, ameliorate atopic dermatitis, and UV-protection (Moon et al., 2020; Nguyen et al., 2021). Meanwhile, CA also showed protective effect against liver damage. Sabitha et al. reported that CA can ameliorate ethanol induced hepatic injury through inhibiting MAPKs and apoptosis signaling via enhancing Nrf2 signaling (Sabitha et al., 2020). Cha et al. reported that CA might protect against acetaminophen induced hepatic injury through inhibiting ROS-dependent apoptosis and inflammation (Cha et al., 2018). In addition, CA can significantly decreased glyoxal or methylglyoxal induced cytotoxicity in isolated rat hepatocytes (Maruf et al., 2015). These results suggested that CA is a promising plant-derived polyphenol against liver damage.

Recently, a study showed that CA can exert radio-protective effect against oxidative damages of liver, spleen, and bone marrow progenitor cells (Kook et al., 2017). Besides, our previous study showed that CA can protect against radiation-induced intestinal injury via inhibition of oxidative stress, inflammatory response and pyroptosis (Li et al., 2021). Since CA is cost effective, higher bioavailability and less toxicity, it might be a good radioprotector against RILD. Hence, this study aimed to test the radioprotective effect of CA in the C57BL/6 mice.

## Materials and methods

### Animals

Male C57BL/6 mice weighing 20–25 g were obtained from Shanghai Jihui Laboratory Animal Co. Ltd. (Shanghai, China), and were housed in a temperature controlled room and a 12-h light/dark cycle. The animals were allowed free access to sterilized water and a standard diet. The animal experiment protocol was approved by the Animal Ethics Committee of Soochow University. All institutional and national guidelines for the care and use of laboratory animals were followed.

### Experimental design

In this study, sixty mice were randomly divided into five groups of 12 mice each. These groups were Control group, IR group, IR + CA50 group, IR + CA100 group and IR + CA200 group. The details were listed below:

**Control group**: Mice received 0.5% carboxymethyl cellulose sodium (1.0 mL/100 g) through gavage administration once daily for 4 consecutive days.

**IR group**: Mice were given 0.5% carboxymethyl cellulose sodium (1.0 mL/100 g) for 4 consecutive days since the day exposed them to a single dose of 4.0 Gy total body X-ray (Simens company, Munich, Germany) irradiation at a dose rate of 2.0 Gy/min. Thirty minutes after the first dose, the mice were exposed to irradiation.

**IR + CA50 group**: Mice were gavage administered with CA (purchased from Sigma-Aldrich company) at the dosage of 50 mg/kg body weight for 4 consecutive days. The other procedures were similar to the IR group.

**IR + CA100 group**: Mice were administered with CA (100 mg/kg body weight) for 4 consecutive days. The other procedures were similar to the IR group.

**IR + CA200 group**: Mice were administered with CA (200 mg/kg body weight) for 4 consecutive days. The other procedures were similar to the IR group.

All the mice were sacrificed on day 3.5 or 14 post-irradiation. Then, the blood and liver tissue were collected for further analysis.

### Liver mass index determination

Before sacrificed, final body mass of the mice was weighed. Immediately after the mice were sacrificed, the liver tissues were collected, weighed and liver mass index was calculated according to the following equation (Galal et al., 2018):

$${\text{Liver}}\,{\text{mass}}\,{\text{index}}\,\left( \% \right) = {\text{Liver}}\,{\text{mass}}\,\left( {\text{g}} \right)/{\text{Body}}\,{\text{mass}}\,\left( {\text{g}} \right) \times {1}00$$

### Hematology analysis and serum liver enzymes detection

About 30 μL of the peripheral blood was put into potassium EDTA collection tube for hematology analysis by a Bayer ADVIA® 2120i Hematology System (Simens company, Munich, Germany). The rest of blood was placed at 4 °C for half an hour, and centrifuged at 3000×*g* at 4 °C for 10 min. To detect the serum alanine aminotransferase (ALT), aspartate aminotransferase (AST) and alkaline phosphatase (AKP) activities, serum was tested using commercial kits (Nanjing Jiancheng Bioengineering Institute, Nanjing, China) according to the manufacturer’s protocol.

### Histopathological analysis

Liver tissues were fixed in 10.0% neutral formalin, sectioned (4–5 μm thickness), stained with hematoxylin and eosin (H&E), and examined under a light microscope (Leica DMi8; Leica Corp., Weztlar, Germany). Images were captured with a digital camera at magnification of × 200.

### Immunochemistry analysis of BAX and Bcl-xL

For immunochemistry analysis, hepatic tissue section slides were incubated with anti-BAX rabbit polyclonal antibody (Servicebio biotechnology corp., Wuhan, China) and anti-Bcl-xL rabbit monoclonal antibody (Abcam, Cambridge, UK). After incubation with the primary antibody overnight at 4 °C, the slides were washed with PBS (pH 7.4) 3 times and incubated with goat anti-rabbit secondary antibody for 50 min under room temperature. Then, the slides were stained with diaminobenzidine tetrahydrochloride and counterstained with hematoxylin for 3 min. After dehydration and mounting, the slides were visualized under a Leica microscope (Leica DMi8; Leica Corp., Weztlar, Germany). The image pro plus 6.0 software (Media Cybernetics, Rockville, MD, USA) was used to analyze the Integrated Optical Density (IOD) and area of the positive cells to determine the relative protein expression ratio (IOD/Area).

### Statistical analysis

Data were expressed as mean ± S.E.M. Statistical analyses were performed using SPSS version 22.0 software, and results were compared using one-way ANOVA followed by LSD post-hoc test. A *p* value of < 0.05 was considered statistically significant.

## Results and discussion

In daily life, the radiation dose was very low, the natural radiation dose was about 350 nGy/h in the U.S (Castillo et al., 2015). Absorbed dose is a measure of how much radiant energy is absorbed by the body, 1 Gy means that 1 kg of tissue absorbs 1 J of energy (1 Gy = 1 J/kg). The effective dose is obtained by multiplying the absorbed dose by coefficients and weighting factors related to the type of radiation and the irradiated organ (Harrison et al., 2016). The unit of effective dose was sievert (Sv, 1 Sv = 1000 mSv = 1 J/kg). The annual global per caput effective dose due to natural radiation sources is 2.4 mSv (Han et al., 2018). The total effective doses of positron emission tomography-computed tomography (PET/CT) might be the highest dose used in medical diagnosis. A previous study from Hong Kong population revealed that the total effective doses of PET/CT was ranged from 13.45 to 32.18 mSv (Huang et al., 2009). In this study, the radiation dose of 4 Gy used only occurred in radiotherapy, experimentation, work in nuclear environment, and accidental radiation releases.

Liver is considered as the most important organ in human body. It plays a crucial role in carbohydrates metabolism, bile production, vitamins storage, and hormones secretion. Besides, it involves in the immune response, blood production, and detoxification (Ali et al., 2020). Radiotherapy for treatment of cancers in the upper abdomen or lower thorax often results in RILD, which might be life-threatening. Therefore, it is important to develop nontoxic compounds to reduce the liver damage of IR.

Bioactive phytochemicals like polyphenols can alleviate RILD. A study revealed that date syrup could alleviate RILD, which might be attributed to presence of proanthocyanidins, anthocyanins, β-carotene and phenolic acids (Abou-Zeid et al., 2018). Besides, persimmon leaf extract, which rich in carotenoids, ferulic acid, protocatechuic acid, vanillic acid and *p*-coumaric acid, showed protective effect against RILD (Ashry et al., 2017). In addition, polyphenols including ferulic acid (Das et al., 2014), crocin (Bakshi et al., 2020) and epigallocatechin gallate (Yi et al., 2020) also exhibited protection against RILD. These results demonstrated that bioactive polyphenols can be used to reduce liver adverse side effects induced by IR. CA is also found as a promising polyphenol that may be a good radioprotector against IR-induced damage. The aim of the present study was to find out the protective role of CA against RILD.

### Effect of CA on the body mass, liver mass and liver mass index of mice

Herein, IR caused weight loss of mice, and was reversed by CA treatment. Tables 1 and 2 showed the final body mass, liver mass and liver mass index of mice in each group. The final body mass showed a slight decrease in IR group on 3.5 day after the irradiation and recovered on 14 day after irradiation. Consequently, the liver mass index revealed a slight increase in IR group compared to the control group on 3.5 day after the irradiation. After CA treatment, the body mass showed a slight increase on 3.5 day after the irradiation, and the liver mass index was significantly reversed in IR + CA100 group as compared with IR group (*p* < 0.05) on 3.5 day after the irradiation. On 14 day after the irradiation, the liver mass index showed no statistical difference in each group. These results showed that irradiation might cause damage to the mice, and CA treatment can improve the life quality of mice to some extent.

**Table 1:** Effect of CA on body and liver mass, as well as hematopoietic function parameters of mice in different groups on day 3.5 after irradiation

Parameters	Control	IR	IR + CA50	IR + CA100	IR + CA200
Final body mass(g)	21.96 ± 0.83	20.42 ± 0.88	21.67 ± 0.46	21.78 ± 0.41	21.40 ± 0.49
Liver mass(g)	1.02 ± 0.07	0.98 ± 0.04	1.02 ± 0.04	0.96 ± 0.02	1.08 ± 0.04
Liver mass index(%)	4.64 ± 0.15	4.82 ± 0.07	4.69 ± 0.07	4.43 ± 0.07^#^	5.05 ± 0.15
WBC(× 10^3^ cells/μL)	4.45 ± 0.28	0.50 ± 0.05^**^	0.33 ± 0.03	0.40 ± 0.05	0.36 ± 0.02
RBC(× 10^6^ cells/μL)	10.01 ± 0.47	9.48 ± 0.30	9.72 ± 0.08	9.90 ± 0.18	9.99 ± 0.15
Hb(g/dL)	13.45 ± 0.66	13.50 ± 0.37	13.70 ± 0.10	13.95 ± 0.25	13.85 ± 0.14
HCT(%)	59.00 ± 2.17	56.48 ± 1.39	56.90 ± 0.71	57.35 ± 0.76	57.80 ± 0.82
PLT(× 10^3^ cells/μL)	1160.00 ± 35.41	1186.50 ± 77.95	1087.00 ± 50.11	1177.50 ± 25.50	1228.00 ± 17.88

*IR* Irradiation, *IR* + *CA50* irradiation + 50 mg/kg CA, *IR* + *CA100* irradiation + 100 mg/kg CA, *IR* + *CA200* irradiation + 200 mg/kg CA. *WBC* white blood cells, *RBC* red blood cells, *Hb* hemoglobin, *HCT* hematocrit, *PLT* platelets

*p < 0.05, **p < 0.01 compared with Control group

^#^*p* < 0.05, ^##^*p* < 0.01 compared with IR group

**Table 2:** Effect of CA on body and liver mass, as well as hematopoietic function parameters of mice in different groups on day 14 after irradiation

Parameters	Control	IR	IR + CA50	IR + CA100	IR + CA200
Final body mass(g)	22.91 ± 0.50	22.37 ± 0.37	22.19 ± 0.52	22.39 ± 0.33	22.04 ± 0.66
Liver mass(g)	1.01 ± 0.02	1.01 ± 0.02	0.98 ± 0.08	1.01 ± 0.02	0.96 ± 0.04
Liver mass index(%)	4.41 ± 0.02	4.53 ± 0.07	4.40 ± 0.29	4.53 ± 0.03	4.37 ± 0.07
WBC(× 10^3^ cells/μL)	5.37 ± 0.63	1.32 ± 0.06^**^	1.48 ± 0.13	2.28 ± 0.26^#^	1.72 ± 0.16
RBC(× 10^6^ cells/μL)	10.23 ± 0.29	7.56 ± 0.18^**^	8.28 ± 0.17^#^	8.46 ± 0.13^##^	8.10 ± 0.16
Hb(g/dL)	16.63 ± 0.27	13.46 ± 0.23^**^	14.40 ± 0.26^##^	14.61 ± 0.20^##^	14.14 ± 0.21^#^
HCT(%)	63.21 ± 2.06	47.01 ± 1.05^**^	50.87 ± 1.02^#^	51.56 ± 0.61^#^	49.11 ± 0.83
PLT(× 10^3^ cells/μL)	1342.86 ± 188.83	1442.86 ± 35.90	1499.43 ± 49.00	1707.14 ± 64.08	1759.43 ± 74.93

*IR* irradiation, *IR* + *CA50* irradiation + 50 mg/kg CA, *IR* + *CA100* irradiation + 100 mg/kg CA, *IR* + *CA200* irradiation + 200 mg/kg CA. *WBC* white blood cells, *RBC* red blood cells, *Hb* hemoglobin, *HCT* hematocrit, *PLT* platelets

*p < 0.05, **p < 0.01 compared with control group

^#^*p* < 0.05, ^##^*p* < 0.01 compared with IR group

### Effect of CA on the hematopoietic function of mice

Hematological system is comprised of the blood, spleen, bone marrow and liver. The hematopoietic system is sensitive to IR. IR would reduce the proliferation and division of blood cells, thus lead to peripheral blood changes. The quantity changes of red blood cells (RBC), white blood cells (WBC) and platelets (PLT) can reflect the functional state of bone marrow hematopoietic tissues (Li et al., 2016; Wang et al., 2020). Development of agents that can attenuate IR-induced hematopoietic injury is also important (Long et al., 2018). Herein, IR caused significant damage to hematopoietic function parameters, and CA treatment markedly improved the hematological damages. The tables (Tables 1 and 2) presented the hematological indicators of mice with and without CA treatment. On day 3.5 and day 14 after irradiation, WBC in IR group decreased significantly as compared with control group (*p* < 0.01). After CA treatment, WBC recovered to some extent, and increased significantly in IR + CA100 group on day 14 after irradiation (*p* < 0.05). On day 14 after irradiation, RBC showed a significant decrease (*p* < 0.01) and it increased significantly in IR + CA50 group (*p* < 0.05) and IR + CA100 group (*p* < 0.01). PLT exhibited no significant difference between control group and IR group on day 3.5 and day 14 after irradiation. After CA treatment, PLT level was increased dependent on CA dose level. On day 14 after irradiation, the PLT level displayed the highest level in IR + CA200 group among all the CA treatment groups. In addition, on day 14 after irradiation, hemoglobin (Hb) and hematocrit (HCT) in IR group exhibited significant decrease as compared with control group (*p* < 0.01). While, on day 14 after irradiation, Hb in all CA treatment groups presented significant increase as compared with IR group, HCT in IR + CA50 group and IR + CA100 group showed significant increase as compared with IR group. Above of all, irradiation induced significant damage to hematological system, CA treatment can rescue the damage to a certain extent. However, CA can not completely remove the damages in a short period. Similarly, even with mouse serum from non-radiated mice injection, the hematological damages induced by 4 Gy total body irradiation cannot completely recovery on 15 day after irradiation (Zhang et al., 2019).

### Effect of CA on the serum liver enzymes of mice

**Fig. 1:**
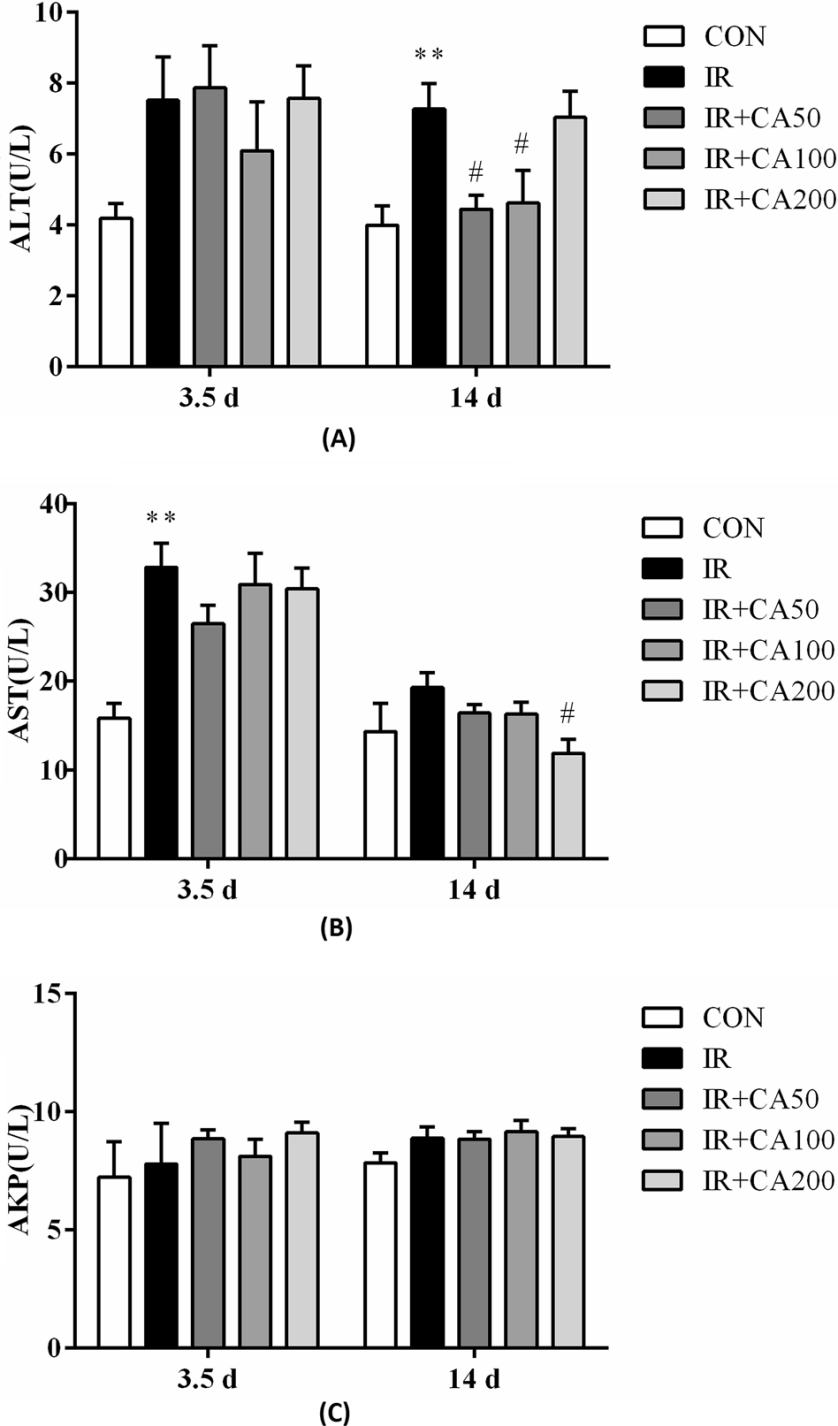
CA influences serum liver enzymes in irradiated mice. **A** ALT activity; **B** AST activity; **C** AKP activity. ALT, AST and AKP activities were determined by using commercial kits. *ALT* alanine aminotransferase, *AST* aspartate aminotransferase, *AKP* alkaline phosphatase, *Control* control group, *IR* ionizing radiation group, *IR + CA50* ionizing radiation + 50 mg/kg body weight of CA group, *IR + CA100* ionizing radiation + 100 mg/kg body weight of CA group, *IR + CA200* ionizing radiation + 200 mg/kg body weight of CA group. Data were expressed as the mean ± S.E.M. *p < 0.05, **p < 0.01 compared with control group; ^#^*p* < 0.05, ^##^*p* < 0.01 compared with IR group

The elevation of serum transaminases like ALT, AST is indicative of hepatocyte damage leading to increase in cell membrane permeability that facilitates transaminases leak out to bloodstream (Pradeep et al., 2012). Currently, an increase in the activity of ALT and AST observed in the serum indicated that the cell membrane ruptured after irradiation. Figure 1 demonstrated the activities of serum ALT, AST and AKP in each experimental group. As compared with control group, the serum ALT activity increased 179.31% and 182.00% (*p* < 0.01) in IR group, respectively on 3.5 day and 14 day after irradiation. After CA treatment, the serum ALT activity significantly decreased (*p* < 0.05) in IR + CA50 group and IR + CA100 group compared to IR group. As for serum AST activity, it increased 207.53% (*p* < 0.01) and 134.99% in IR group relative to control group, respectively on 3.5 day and 14 day after irradiation. After CA treatment, serum AST activity decreased compared to IR group. Besides, it showed significantly reduction in IR + CA200 group compared to IR group. In addition, serum AKP activity exhibited no significant difference among various groups on day 3.5 and day 14 after irradiation.

Treatment with CA decreased the activities of ALT and AST in serum, suggesting that CA might offer radioprotection through maintaining the hepatocyte membrane integrity. Previous studies also revealed that CA (50 mg/kg body weight) can reduce serum ALT and AST activity in ethanol induced hepatic injury in rats and acetaminophen-induced hepatotoxicity in mice (Cha et al., 2018; Sabitha et al., 2020).

### Effect of CA on the histopathological analysis

**Fig. 2:**
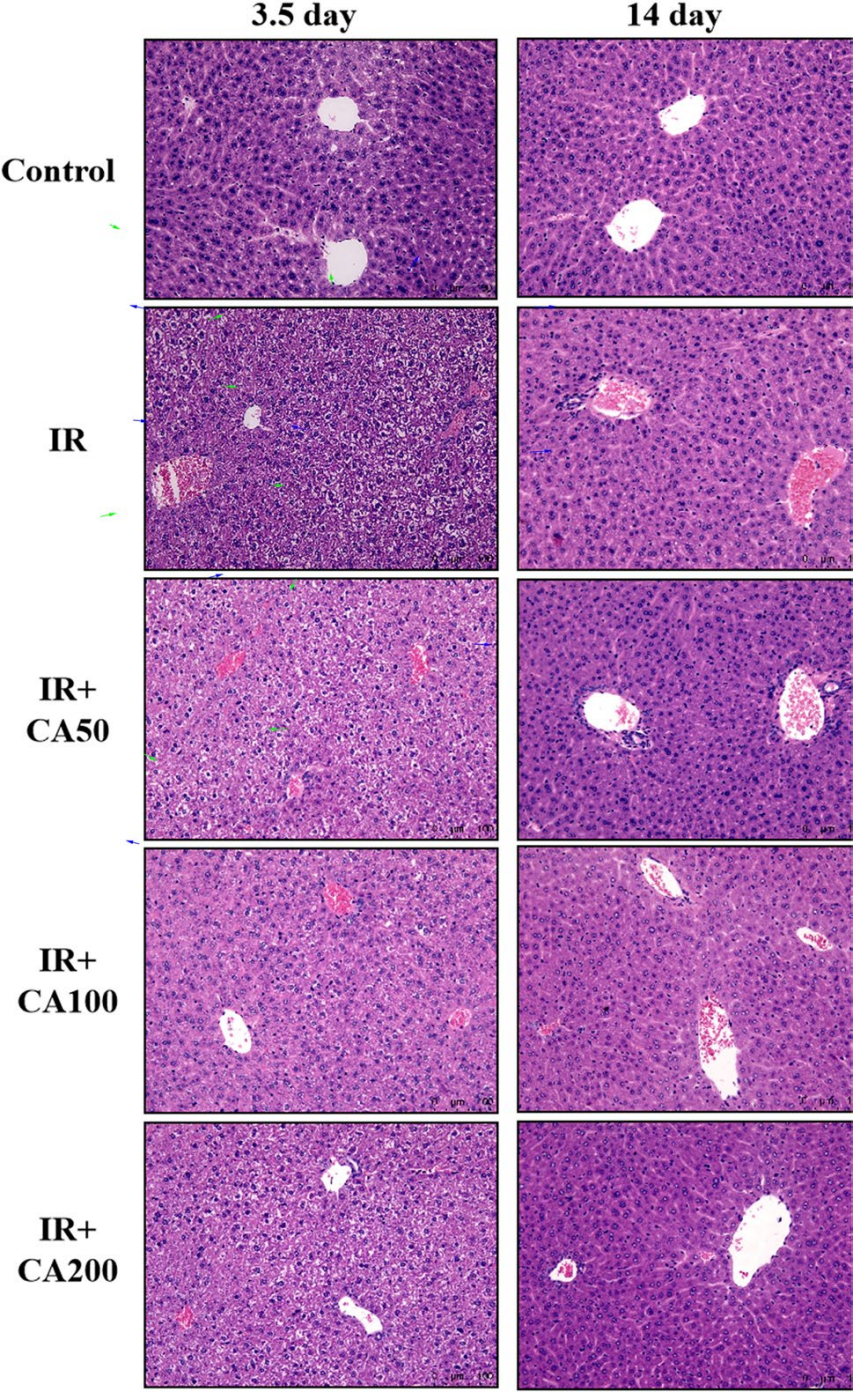
Representative H&E-stained images showing the liver morphology (200 ×). The blue arrow referred to portal vein congestion. Besides, the green arrow referred to hepatocellular swelling, karyopyknosis, and steatosis. *H&E* hematoxylin and eosin; *Control* control group, *IR* ionizing radiation group, *IR* + *CA50* ionizing radiation + 50 mg/kg body weight of CA group, *IR* + *CA100* ionizing radiation + 100 mg/kg body weight of CA group, *IR* + *CA200* ionizing radiation + 200 mg/kg body weight of CA group

Total body exposure of male mice to 4 Gy X-ray irradiation has provoked significant damage to liver tissue, as evidenced by histopathological analysis. IR induced significant hepatic sinusoidal congestion and steatosis, and it was ameliorated by CA treatment. Figure 2 exhibited the histopathological results of hepatic tissue in each group. The hepatic tissue of control group showed normal histological structure. The liver cells in control groups contained a large, round nucleus, and a granular acidophilic cytoplasm. However, on 3.5 day after irradiation, the hepatic structure of IR group showed hepatocellular swelling, partly karyopyknosis, cytoplasmic vacuolation, and portal vein congestion with fibrinoid necrosis. The blue arrows in Fig. 2 referred to portal vein congestion and dilation. Besides, the green arrows referred to hepatocellular swelling, karyopyknosis, and steatosis. Similarly, Abdel-Magied et al. also found portal vein congestion with fibrinoid necrosis, hepatocellular swelling and expanding occurred in rats exposed to irradiation (Abdel-Magied et al., 2019). These histopathological changes might induce hepatic necrosis changes and abnormal liver function, which were consistent with the serum liver enzymes changes. On 14 day after irradiation, the hepatic structure restored to a certain extent. Besides, the sinusoidal congestion and steatosis score for IR groups were significantly increased (Fig. 5A). These histopathological changes were improved in CA treatment groups. Specifically, the sinusoidal congestion and steatosis score of IR + CA100 group was significantly decreased compared to IR group on 3.5 day after irradiation. Meanwhile, the sinusoidal congestion and steatosis score of IR + CA200 group was significantly decreased compared to IR group on 14 day after irradiation.

Similarly, Ekinci Akdemir et al. showed that CA treatment (100 mg/kg body weight) can improve hydropic degeneration, vascular congestion, sinusoidal dilatation in acute liver damage induced by Cisplatin (Ekinci et al., 2017). With regard to hepatic steatosis, previous studies also found out that CA can inhibit liver fat accumulation. Lee et al. showed that CA can decrease the hepatic lipid droplet accumulation in rats given a high cholesterol diet (Lee et al., 2003). Sabitha et al. also revealed that CA can inhibit steatosis in ethanol exposed rat liver tissue (Sabitha et al., 2020). In vitro studies demonstrated that CA might protect against hepatic steatosis by upregulating CPT-1 expression, inhibiting COX-2 expression and PGE2 accumulation (Xie et al., 2014; Yan et al., 2020).

### Effect of CA on the BAX and Bcl-xL levels in hepatic tissues

**Fig. 3:**
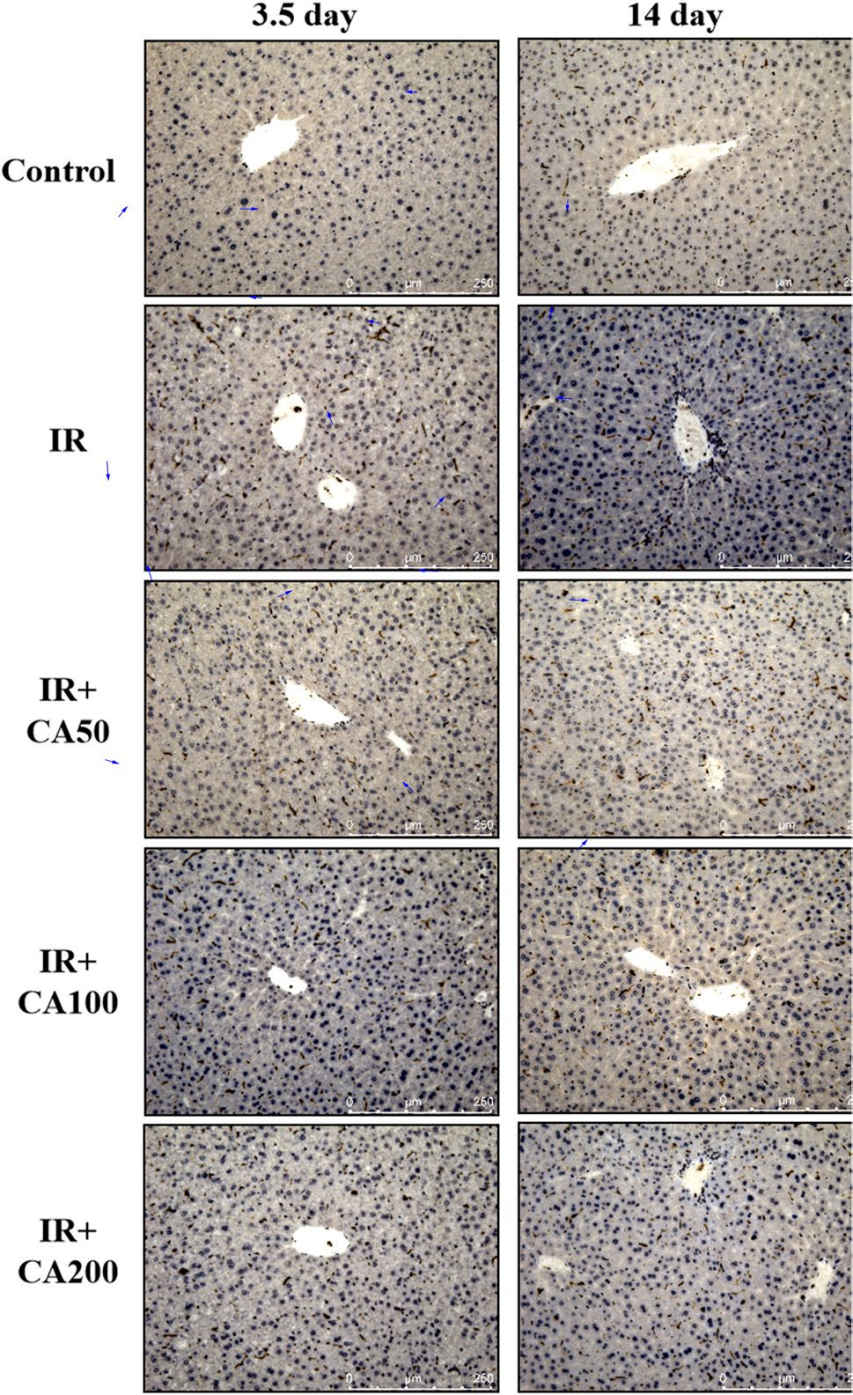
Representative immunohistochemistry images of BAX in liver tissues (200 ×). The BAX positive area was in brown color and indicated in blue arrows. *Control* control group, *IR* ionizing radiation group, *IR* + *CA50* ionizing radiation + 50 mg/kg body weight of CA group, *IR* + *CA100* ionizing radiation + 100 mg/kg body weight of CA group, *IR* + *CA200* ionizing radiation + 200 mg/kg body weight of CA group

**Fig. 4:**
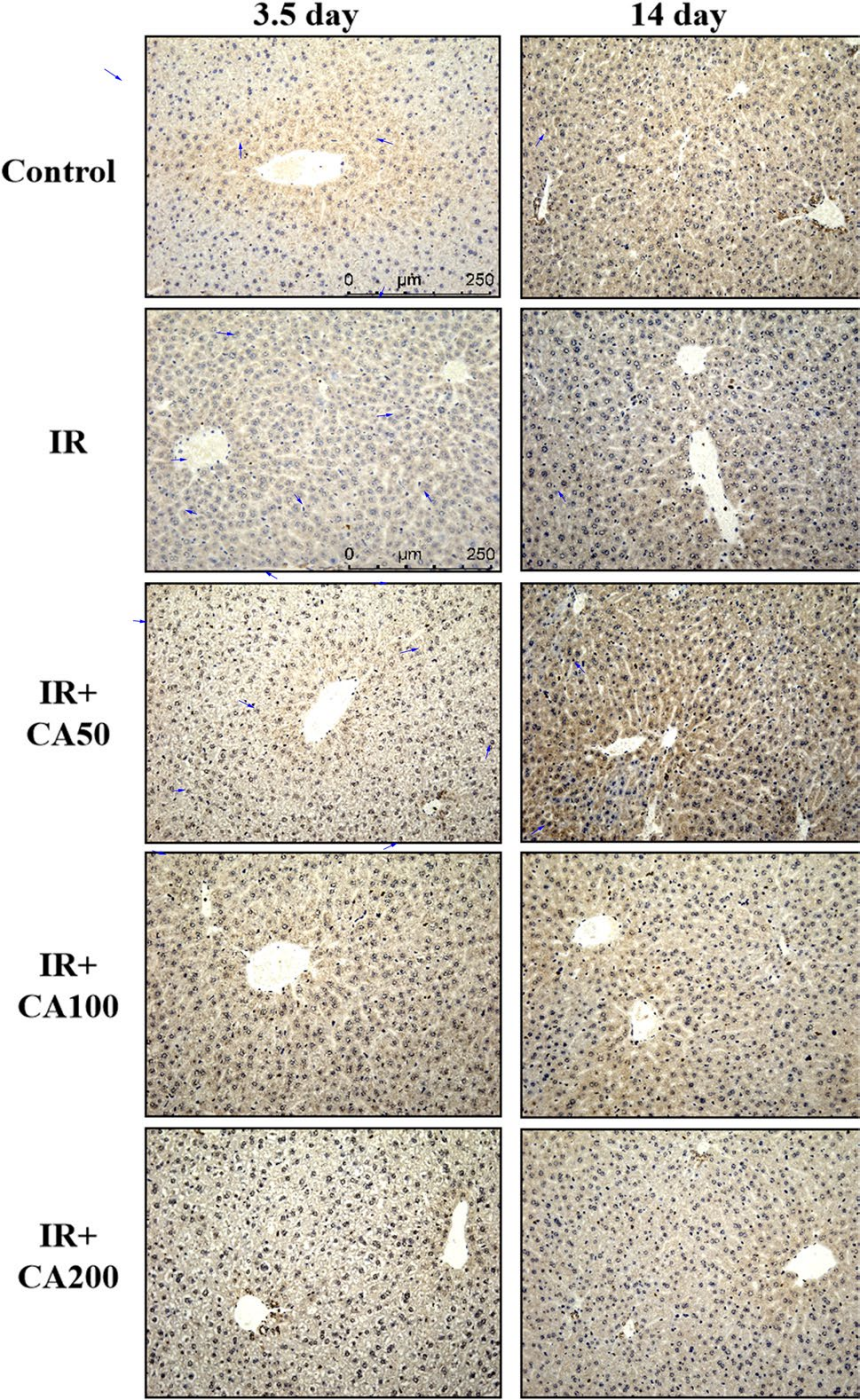
Representative immunohistochemistry images of Bcl-xL in liver tissues (200 ×). The Bcl-xL positive area was in brown color and indicated in blue arrows. *Control* control group, *IR* ionizing radiation group, *IR* + *CA50* ionizing radiation + 50 mg/kg body weight of CA group, *IR* + *CA100* ionizing radiation + 100 mg/kg body weight of CA group, *IR* + *CA200* ionizing radiation + 200 mg/kg body weight of CA group

**Fig. 5:**
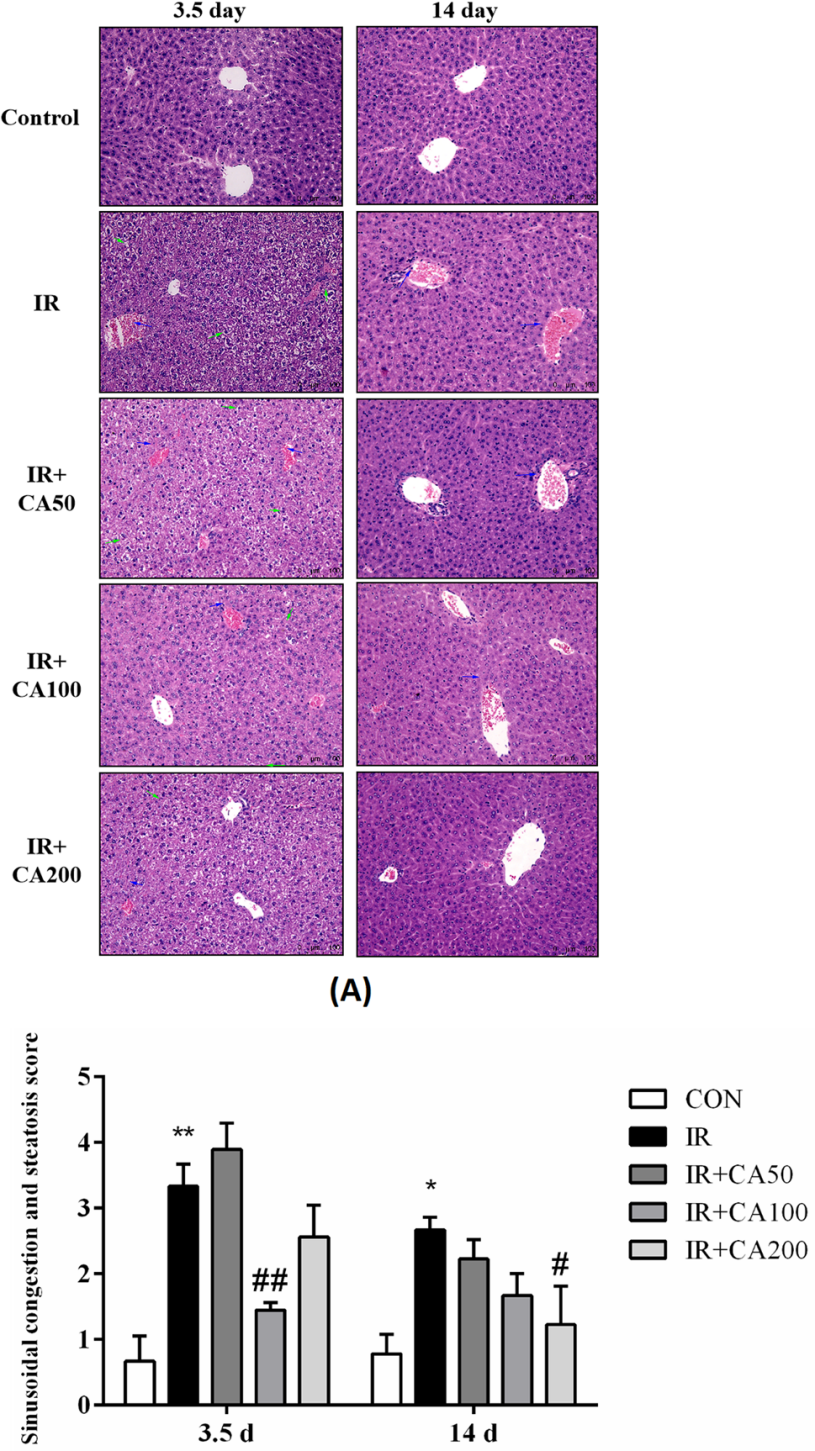
CA ameliorates liver damage in irradiated mice. **A** The sinusoidal congestion and steatosis score of radiation-induced liver damage after radiation. **B** The relative expression ratio of BAX in liver tissues. **C** The relative expression ratio of Bcl-xL in liver tissues. The levels of BAX and Bcl-xL protein expression were analyzed after normalization to that of control group. *Control* control group, *IR* ionizing radiation group, *IR* + *CA50* ionizing radiation + 50 mg/kg body weight of CA group, *IR* + *CA100* ionizing radiation + 100 mg/kg body weight of CA group, *IR* + *CA200* ionizing radiation + 200 mg/kg body weight of CA group. Data were expressed as the mean ± S.E.M. *p < 0.05, **p < 0.01 compared with control group; ^#^*p* < 0.05, ^##^*p* < 0.01 compared with IR group

In addition, irradiation would cause apoptotic cellular damages through activating proapoptotic molecules, and suppressing antiapoptotic molecules (Kim et al., 2017). BAX is a pro-apoptotic protein, and BcL-xL is an antiapoptotic protein. Immunochemistry experiments (Fig. 3) showed that irradiation induced BAX expression in the liver tissues, and was suppressed after CA treatment on day 14 after irradiation. The relative BAX expression ratio of IR + CA200 group was significantly decreased compared to IR group on 14 day after irradiation (Fig. 5B). As for Bcl-xL (Figs. 4 and 5C), its relative expression ratio was significantly decreased on day 3.5 after irradiation. After CA treatment, no significant differences were observed as compared with IR group on day 3.5 and day 14 after irradiation. As CA treatment could suppress BAX expression, thus it might protect against hepatocyte apoptosis. Sabitha et al. also proved that CA can protect ethanol induced apoptosis by inhibiting the expression of BAX and caspases in liver tissue (Sabitha et al., 2020). Cha et al. concluded that CA might protect against acetaminophen-induced hepatotoxicity by inhibition of ROS-dependent hepatic apoptosis (Cha et al., 2018). Parvizi et al. summarized that CA showed hepatoprotective effect in a rat model of ischemia–reperfusion by down-regulating caspase-3, which is an apoptotic gene protein (Parvizi et al., 2020). These studies all supported that CA can protect against hepatocyte apoptosis.

### The optimum dose of CA against RILD

Kook et al. reported that oral administration of CA (20 mg/kg body weight) can prevent irradiation induced liver damages including reduction of serum ALT and AST activities, elevation of liver anti-oxidant enzyme activities, and inhibition of Nrf2 expression (Kook et al., 2017). Herein, as compared with doses of 50 mg/kg body weight and 200 mg/kg body weight, 100 mg/kg body weight of CA showed better protective effect against RILD. Besides, Sharma et al. reported that 100 mg/kg body weight of CA exhibited a significant optimum effect against 1,2 dimethylhydrazine induced colonic preneoplastic lesions compared to doses of 50 mg/kg body weight and 200 mg/kg body weight (Sharma et al., 2017). Other studies also suggested that 100 mg/kg body weight of CA might be the optimum dose to protect against lipopolysaccharide-induced lung inflammation, and hypoxia-induced pulmonary and cerebral edema (Kheiry et al., 2019; Li et al., 2018, 2019). Since CA showed low toxicity in mice (LD50 = 2850 mg/kg body weight) (Li et al., 2021), it is a promising natural plant-derived compound to prevent RILD.

In summary, the present study also showed oral administration of CA significantly attenuates RILD. It can inhibit IR-induced hepatic sinusoidal congestion and steatosis, reduce BAX protein expression, reverse serum ALT and AST activities change and improve hematopoietic function. Future studies need to study the mechanism of CA in providing radioprotection against liver damage. Based on the above results, CA might be a promising natural compound against RILD.
