# Correction to: Amelioration of radiation‑induced liver damage by *p*-coumaric acid in mice

**DOI:** 10.1007/s10068-023-01290-5

**Published:** 2023-03-21

**Authors:** Yun-Hong Li, Jiang-Xue Wu, Qian He, Jia Gu, Lin Zhang, Hao-Zhi Niu, Xin-Wen Zhang, Han-Ting Zhao, Jia-Ying Xu, Li-Qiang Qin

**Affiliations:** 1 Department of Nutrition and Food Hygiene, School of Public Health, Soochow University, 199 Renai Road, Suzhou, 215123 Jiangsu Province China; 2 State Key Laboratory of Radiation Medicine and Protection, School for Radiological and Interdisciplinary Sciences (RAD-X) Collaborative Innovation Center of Radiation Medicine of Jiangsu Higher Education Institutions, Soochow University, 199 Renai Road, Suzhou, 215123 Jiangsu Province China

**Correction to: Food Sci Biotechnol (2022) 31:1315-1323** https://doi.org/10.1007/s10068-022-01118-8

In the original publication, incorrect versions of Figs. 2, 3, 4 and 5 were published. Specifically, the arrows in Figs. 2, 3 and 4 were moved outside the representative images, and the Fig. 5 was wrongly replaced by another figure. The correct version of Figs. 2, 3, 4, and 5, were shown below.

**Fig. 2:**
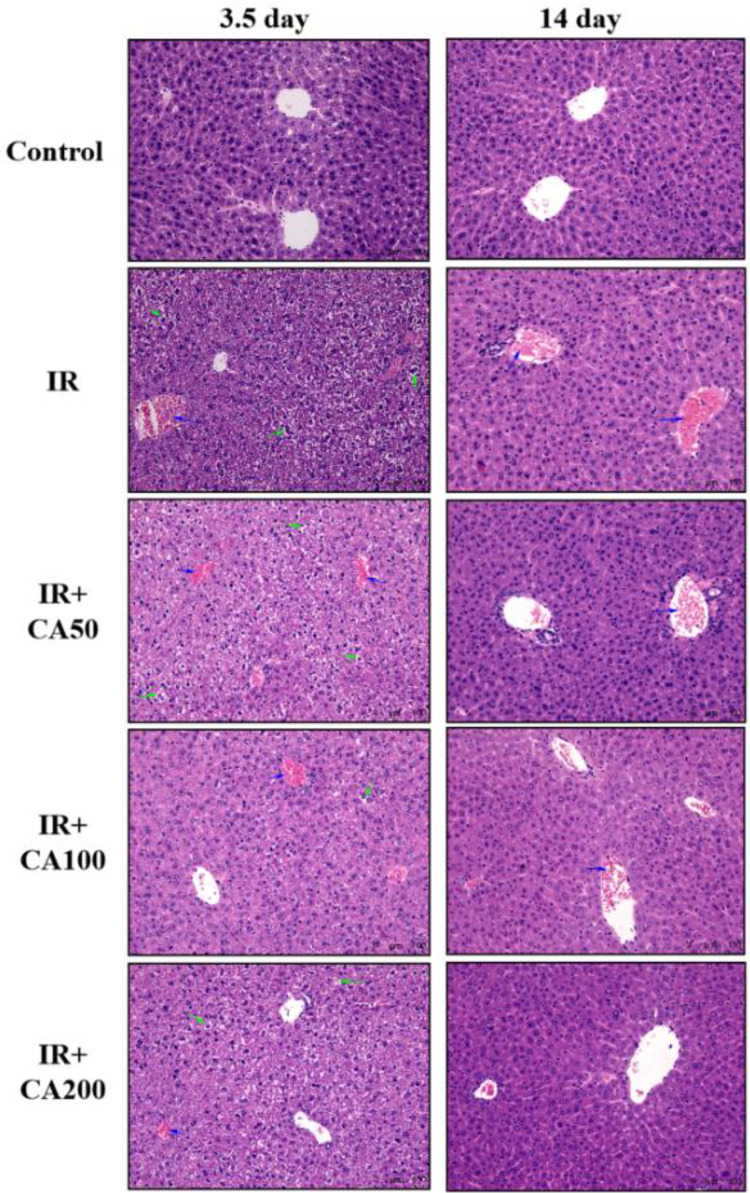
Representative H&E-stained images showing the liver morphology (200 ×). The blue arrow referred to portal vein congestion. Besides, the green arrow referred to hepatocellular swelling, karyopyknosis, and steatosis. *H&E* hematoxylin and eosin; *Control* control group, *IR* ionizing radiation group, *IR* + *CA50* ionizing radiation + 50 mg/kg body weight of CA group, *IR* + *CA100* ionizing radiation + 100 mg/kg body weight of CA group, *IR* + *CA200* ionizing radiation + 200 mg/kg body weight of CA group

**Fig. 3:**
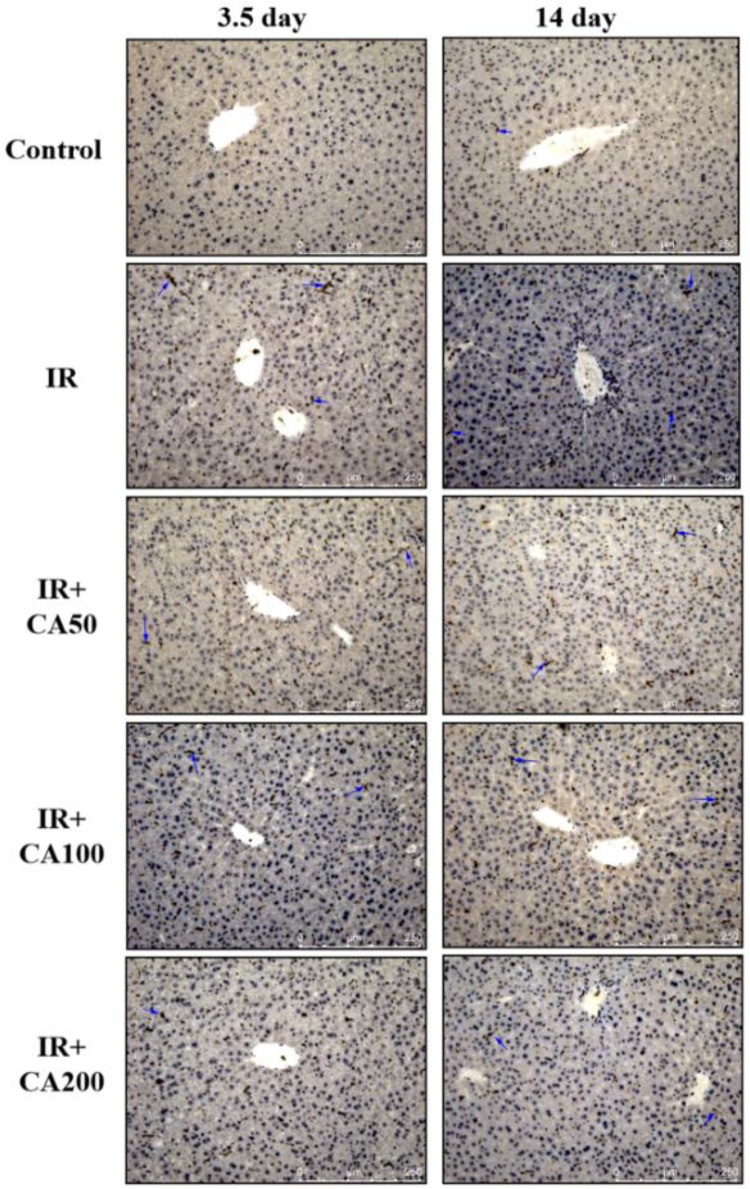
Representative immunohistochemistry images of BAX in liver tissues (200 ×). The BAX positive area was in brown color and indicated in blue arrows. *Control* control group, *IR* ionizing radiation group, *IR* + *CA50* ionizing radiation + 50 mg/kg body weight of CA group, *IR* + *CA100* ionizing radiation + 100 mg/kg body weight of CA group, *IR* + *CA200* ionizing radiation + 200 mg/kg body weight of CA group

**Fig. 4:**
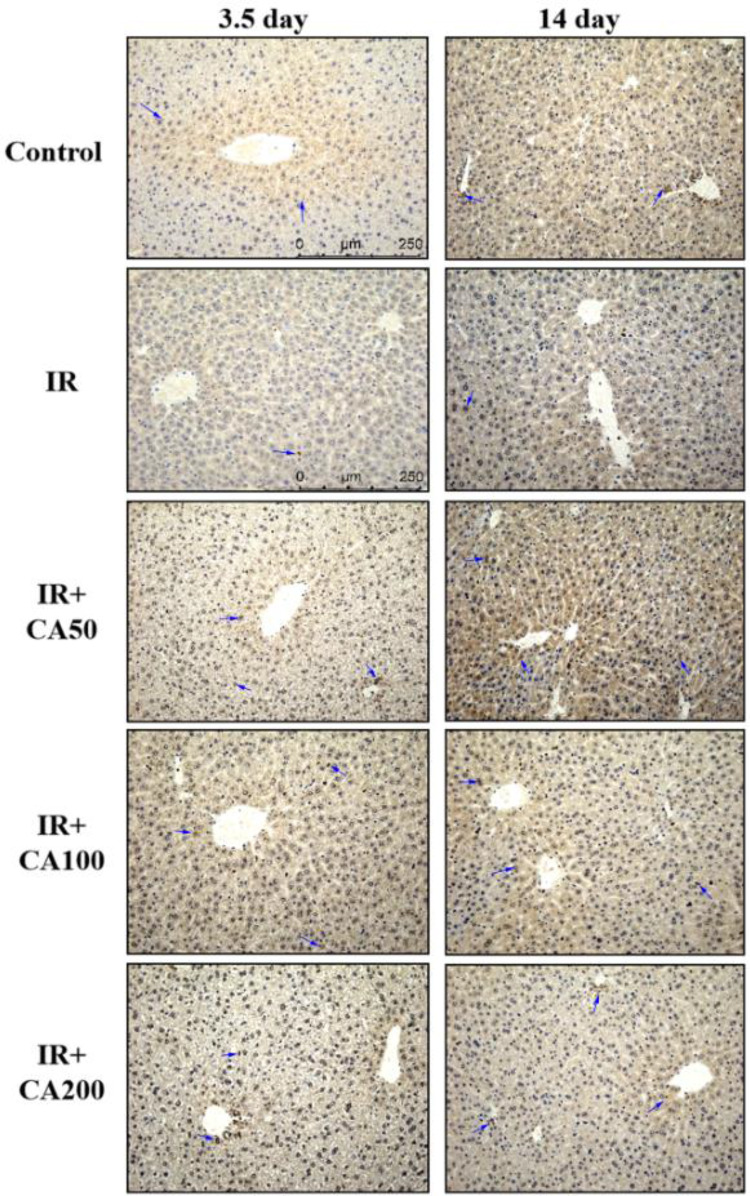
Representative immunohistochemistry images of Bcl-xL in liver tissues (200 ×). The Bcl-xL positive area was in brown color and indicated in blue arrows. *Control* control group, *IR* ionizing radiation group, *IR* + *CA50* ionizing radiation + 50 mg/kg body weight of CA group, *IR* + *CA100* ionizing radiation + 100 mg/kg body weight of CA group, *IR* + *CA200* ionizing radiation + 200 mg/kg body weight of CA group

**Fig. 5:**
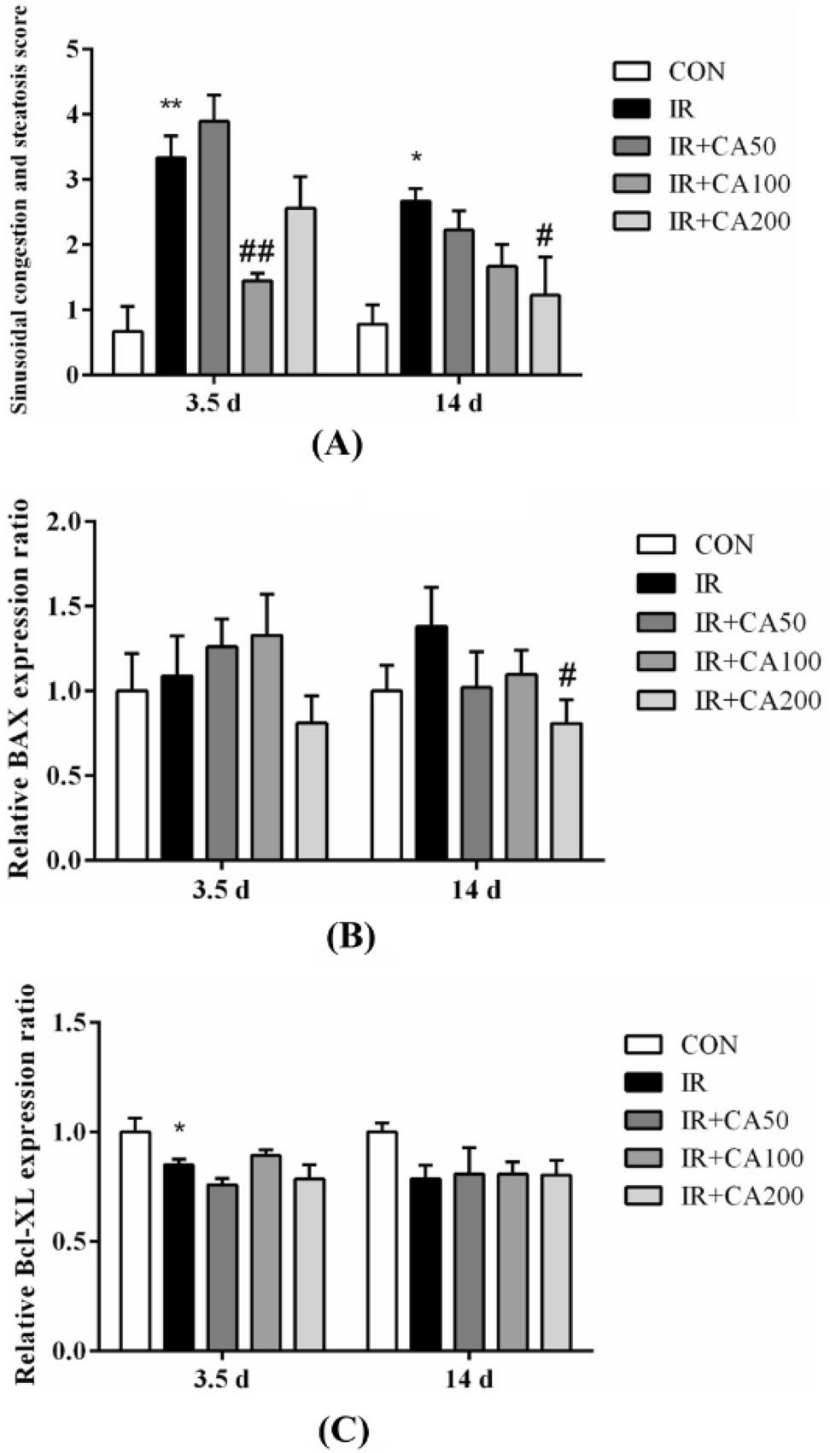
CA ameliorates liver damage in irradiated mice. **A** The sinusoidal congestion and steatosis score of radiation-induced liver damage after radiation. **B** The relative expression ratio of BAX in liver tissues. **C** The relative expression ratio of Bcl-xL in liver tissues. The levels of BAX and Bcl-xL protein expression were analyzed after normalization to that of control group. *Control* control group, *IR* ionizing radiation group, *IR* + *CA50* ionizing radiation + 50 mg/kg body weight of CA group, *IR* + *CA100* ionizing radiation + 100 mg/kg body weight of CA group, *IR* + *CA200* ionizing radiation + 200 mg/kg body weight of CA group. Data were expressed as the mean ± S.E.M. *p < 0.05, **p < 0.01 compared with control group; ^#^*p* < 0.05, ^##^*p* < 0.01 compared with IR group

